# Stabilizers as a design tool for new forms of the Lechner-Hauke-Zoller annealer

**DOI:** 10.1126/sciadv.1601246

**Published:** 2016-10-21

**Authors:** Andrea Rocchetto, Simon C. Benjamin, Ying Li

**Affiliations:** Department of Materials, University of Oxford, Parks Road, Oxford OX1 3PH, U.K.

## Abstract

In a recent paper, Lechner, Hauke, and Zoller (LHZ) described a means to translate a Hamiltonian of *N* spin-^1^/_2_ particles with “all-to-all” interactions into a larger physical lattice with only on-site energies and local parity constraints. LHZ used this mapping to propose a novel form of quantum annealing. We provide a stabilizer-based formulation within which we can describe both this prior approach and a wide variety of variants. Examples include a triangular array supporting all-to-all connectivity as well as arrangements requiring only 2*N* or *N* log *N* spins but providing interesting bespoke connectivities. Further examples show that arbitrarily high-order logical terms can be efficiently realized, even in a strictly two-dimensional layout. Our stabilizers can correspond to either even-parity constraints, as in the LHZ proposal, or odd-parity constraints. Considering the latter option applied to the original LHZ layout, we note that it may simplify the physical realization because the required ancillas are only spin-^1^/_2_ systems (that is, qubits rather than qutrits); moreover, the interactions are very simple. We make a preliminary assessment of the impact of these design choices by simulating small (few-qubit) systems; we find some indications that the new variant may maintain a larger minimum energy gap during the annealing process.

## Teaser

A new formalism to understand a novel class of quantum annealing devices and new designs obtained using the formalism.

## INTRODUCTION

Quantum annealing (QA) is an approach to solving optimization problems, a family of tasks that includes many important examples ranging from financial portfolio management to vehicle routing (*1*, *2*). Typically, the task can be thought of as minimizing a cost function that depends on many variables. In QA, this is done by considering a physical system whose energy corresponds to the cost and by seeking that system’s ground state. One can implement the QA approach using conventional hardware by running an algorithm that mimics quantum behavior (*3*); this is thus a variant of classical simulated annealing (*4*). Alternatively, one can aim to construct real quantum technologies whose components are quantum entities capable of superposition and entanglement. The annealing process can exploit the adiabatic theorem to remain in, or near, the system’s ground state when sufficiently slowly changing parameters (such as a global field). By starting from a Hamiltonian whose ground state can be reliably achieved and morphing slowly into a final Hamiltonian whose energies reflect the cost in the optimization problem, the hope is that measuring the final state reveals a low-cost solution.

There are many interesting questions associated with this approach. The prospects of reaching the ground state of the final Hamiltonian will depend on the rate at which the Hamiltonian is changed versus the size of the gap from the ground state to the lowest excited states; the smaller the gap, the slower the evolution must be (*5*). However, because the gap size cannot be precomputed for problems of meaningful size, it is difficult to be definite about how fast the system can be permitted to evolve or whether the approach can succeed at all given finite temperatures. Thus, the performance of a QA system is hard to predict analytically. Prototype systems have been produced by D-Wave Systems, and several studies have sought to evaluate the power of these systems by empirical testing [see, for example, Rønnow *et al*. (*6*)]. One can also make comparisons with QA simulated by quantum Monte Carlo, although this must be done with caution because there are subtleties with the discretization of time (*7*).

Another important question is that of connectivity. In contrast to conventional computers and circuit model quantum computers, the adiabatic approach involves keeping interactions between qubits “always on” to maintain the energy gap. This implies that the qubits, or spins as we will henceforth refer to them, should continuously maintain direct physical interactions. Ideally, one might wish to be able to connect any physical spin in the device to any other, but in practice, this is inconsistent with interactions that are implemented through short-range physical links. In the D-Wave chips, the set of permitted nonzero links between the physical spins is called the Chimera graph [see Bunyk *et al*. (*8*)]. It is locally rich, but on the large scale, it has the form of a two-dimensional (2D), nearest-neighbor lattice. Typically for a real-world optimization problem, such as the satisfiability problems (*9*, *10*), one would not expect that the variables are tensioned against each other in a pattern that respects any particular geometry. Therefore, for the logical problem to be realized as a physical annealing task, it must be reexpressed in some way.

One solution is based on minor embedding (*11*–*14*): In effect, groups of physical spins are bound together with very strong interactions to form extended single-spin entities. These larger entities have correspondingly more connections to one another. To achieve all-to-all connectivity in this way, the *N* spins of the logical problem must be encoded into Order(*N*^2^) physical spins. However, even assuming that this cost is permissible (and one should expect that the increased number of physical spins corresponds to a reduction in the crucial energy gap), there is the question of whether this approach is practical. When a large number of physical spins are bound together with achievable interaction strengths, it is not clear whether the extended objects will function as equivalent to single logical spins.

An alternative formulation of the mapping was recently proposed by Lechner, Hauke, and Zoller (LHZ) (*15*). According to this approach, the physical spins now represent the links, or relative orientations, between the logical spins. Thus, there is one physical spin whose role is to represent the relative orientation of logical spins 1 and 2: If they are aligned (↑↑ or ↓↓), then the physical spin will take one value (say ↑), whereas if the logical spins are antialigned (↓↑ or ↑↓), then the physical spin takes the opposite orientation (↓). Because there are *N*(*N* − 1)/2 possible pairings of the *N* logical spins, this leads to the same Order(*N*^2^) resource cost as the minor embedding approach; however, one avoids the need to bind multiple physical spins into single entities, and the coupling strengths in the logical model conveniently map to single-spin energies in the physical hardware. We note that a paper by Albash *et al*. (*16*), which makes a detailed comparison of minor embedding versus the LHZ approach, has been posted very recently.

Here, we view the LHZ approach within a stabilizer formalism. By means of this general framework, we introduce three new features that significantly extend the potential applications and implementations of the LHZ annealer.

Our analysis begins by noting that the parity constraints of the original LHZ construction correspond to a set of stabilizers. Each stabilizer is a product of physical *z* operators, which we can constrain to one of its eigenvalues, either +1 (even parity) or −1 (odd parity). After recovering the original LHZ construction, we introduce three generalizations: first, a new layout that makes use of all odd-parity constraints. Such a device can make use of a simpler ancillary structure that should prove easier to implement and, as our simple numerical simulations indicate, might present less frequent level crossings. Second, we show that the stabilizer formalism can be used to discover more efficient physical architectures when some of the couplings in the logical spin system are zero. This is significant given that certain NP (nondeterministic, polynomial time) problems, such as graph coloring, are most challenging to solve when their connectivity graph is not complete (*17*, *18*). Whereas the LHZ paper (*15*) established the principle that physical spins can be removed when the logical model has certain simple restrictions, here, we identify general principles that can permit one to tailor a “bespoke” architecture for a given task. We provide examples where 2*N* − 1 and *N* log *N* physical spins realize nontrivial connectivities between *N* logical spins. Finally, our third generalization introduces structures that encode arbitrarily high-order terms in the logical Hamiltonian (such as $\sigma_{i}^{Z}\sigma_{j}^{Z}\ldots \sigma_{m}^{Z}$) as individual physical spins in a simple 2D layout [in Lechner *et al*. (*15*), three-body terms were realized by moving to a 3D lattice].

Our approach is conceptually straightforward. We take a candidate layout of *N*_P_ physical spins, and we specify *N*_S_ = *N*_P_ − *N* stabilizer constraints. We then nominate *N* of the physical spins, each of which will correspond to a logical spin in the following sense: The *z* operator of a physical spin is identified with the same operator on the logical spin. Finally, we identify the logical *x* operators that are implied by these earlier choices; each will be a product of operators forming a chain that crosses the layout, rather analogously to logical operators in topological codes such as Kitaev’s surface code (*19*). Intersections between these logical *x* chains allow us to find the meaning of each individual physical spin, that is, to identify what product of logical *z* spins it represents.

## RESULTS

### Parity constraint annealing and stabilizer code

We define the logical spin glass model, in which each spin can have an interaction with every other spin as well as an arbitrary local field, as follows

$$H_{\text{logic}}=\sum_{i=1}^{N}h_{i}\sigma_{i}^{Z}+\sum_{i=1}^{N-1}\sum_{j=i+1}^{N}J_{i,j}\sigma_{i}^{Z}\sigma_{j}^{Z}$$ (1)

Note that this Hamiltonian is general in the sense that the local fields *h*_*i*_ and the interaction strengths *J*_*i*,*j*_ can take any value. However, it does not contain three-body or higher interaction terms, which would be convenient for optimizing functions containing terms with three or more variables involved. In the final part of the analysis presented here, we will extend our considerations to logical Hamiltonians that do contain arbitrarily higher-order terms. For simplicity, we will now focus on the case where the logical Hamiltonian has the form given above.

**Fig. 1:**
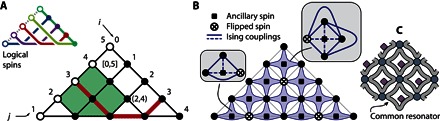
Illustration of the fully connected architecture in the stabilizer formalism. The lattice of ***N*(*N* + 1)/2** physical spins for encoding a fully connected Ising Hamiltonian, here shown for the case of *****N* = 5**** logical spins. (**A**) The larger triangle represents the formal scheme. Black and white dots are the physical spins; the spin at each black dot corresponds to a two-body coupling between logical spins, whereas that at each white dot corresponds to the local field acting on a logical spin. In the small triangle (top left), we show the chains of logical *x* operators (one color for each logical spin). The intersection of two such chains determines the meaning of the individual physical spin at that location. Rounded brackets (…) indicate a physical spin, whereas square brackets […] identify a plaquette; indices *i* and *j* are consistent with those in the main text, for example, in Eq. 3. (**B**) A physical implementation using ancilla spins to realize the stabilizer constraints. In the insets, the lines indicate the Ising couplings between the spins; all solid lines have a common strength, as do all dashed lines. (**C**) A schematic indicating that common resonators might mediate the interactions, similar to the proposal in Fig. 2 of Chancellor *et al*. (*20*).

The task now is to successfully emulate the physics of this ideal, logical Hamiltonian using real architecture in which a larger number of physical spins interact only locally. We begin by selecting a layout for the physical spins; our first choice will be the 2D lattice with a square unit cell as proposed by LHZ (see Fig. 1). This structure contains *N*(*N* + 1)/2 physical spins; therefore, its full Hilbert space is vastly greater than that of the logical Hamiltonian: We must apply constraints to define a suitable subspace. We will specify a set of mutually commuting stabilizers, each being an operator formed by a product of single-spin Pauli operators. We will require that the state of the system be a mutual eigenstate of all these stabilizers with a specified eigenvalue in each case: either +1 or −1. (Note that this is a slight departure from the usual convention where all stabilizer eigenvalues are +1 because any negative value is absorbed into the definition of the stabilizer itself.) As we enforce each such stabilizer, we will halve the dimension of the compatible Hilbert space. Therefore, we will require *N*(*N* + 1)/2 − *N* = *N*(*N* − 1)/2 stabilizers so that the compliant subspace has the desired dimension, 2^*N*^. Our remaining task will be to identify observables in the physical lattice that correspond to the measurement of single spins in the logical Hamiltonian and establish the conditions under which the correspondence is correct.

As shown in Fig. 1A, the physical layout is a lattice of square cells forming a triangular shape. The lattice is composed of *N*(*N* + 1)/2 spins. Figure 1B also introduces the use of additional spins called ancillas, which will be required to enforce the stabilizer constraints in a physically natural way. Their role and structure will be discussed in more detail in the next section. Here, we simply note that when we allude to physical spins without explicitly using the term “ancilla,” we are referring to the *N*(*N* + 1)/2 spins that form the direct physical embodiment. We label each physical spin with two coordinates (*i*, *j*) and each plaquette according to the spin at the top corner; for example, the top plaquette is labeled as [0, 5] in Fig. 1. The reason for this labeling will become apparent; for now, it is simply a systematic way to uniquely tag each spin.

For this formulation to be consistent with the LHZ approach, we select a family of stabilizers that can constrain the parity of the physical spins around each plaquette of the lattice. Plaquette [*i*, *j*] corresponds to a stabilizer

$$S_{\left[i,j\right]}=\{\begin{array}{cc} {\tilde{\sigma }}_{\left(i,j\right)}^{Z}{\tilde{\sigma }}_{\left(i,j-1\right)}^{Z}{\tilde{\sigma }}_{\left(i+1,j\right)}^{Z}{\tilde{\sigma }}_{\left(i+1,j-1\right)}^{Z} & \text{if}\;i+2<j\\ {\tilde{\sigma }}_{\left(i,j\right)}^{Z}{\tilde{\sigma }}_{\left(i,j-1\right)}^{Z}{\tilde{\sigma }}_{\left(i+1,j\right)}^{Z} & \text{if}\;i+2=j \end{array}$$ (2)

where the second case *i* + 2 = *j* simply corresponds to the triangular plaquettes along the base of the lattice. The tilde symbol is used to indicate that the sigma operators $\tilde{\sigma }$ apply to physical spins. Each stabilizer is thus the product of Pauli operators acting on the physical spins around the plaquette. Because each Pauli operator has eigenvalues ± 1 corresponding to its spin being oriented in the positive/negative *z* direction, it follows that the stabilizer’s eigenvalues are ± 1. This value can be interpreted as a parity if we take + 1 to indicate “even” and −1 to indicate “odd” in terms of the number of spins aligned along the negative *z* direction. This odd/even label is natural for four-body stabilizers but can be less intuitive for other stabilizers—therefore, we will use the term “odd/even” only for the four-body case, and more generally, we will speak of positive and negative stabilizers.

We will require that the states of interest |*L*〉 be good eigenstates of all these stabilizers, that is

$$S_{\left[i,j\right]}|L\rangle =\nu_{i,j}|L\rangle$$

where we will specify, for every plaquette [*i*, *j*], each individual ν_*i*,*j*_ as + 1 or −1. Equivalently, writing *P*_0_ as the projector into the legitimate subspace, we require *S*_[*i*,*j*]_*P*_0_ = ν_*i*,*j*_*P*_0_. We easily verify that this set of stabilizers mutually commute (all are *z* basis operators) and, moreover, that they are independent: Specifying the eigenvalues of any subset does not constrain the eigenvalues of the remaining ones. This latter property is confirmed by noting that as we consider each new plaquette of the lattice, we are encountering at least one new qubit. (Note that, in contrast, a feature of the 2D toric stabilizer code us that specifying all but one of the stabilizers in a given basis will logically imply the value of the last one.) It is worth remarking that the legitimate states of the physical spins are formally stabilizer states if we opt to absorb the ν_*i*,*j*_ factors into the stabilizers, but we find it convenient to regard the stabilizers as fixed entities and ν_*i*,*j*_ as their required eigenvalues.

The observable properties of the physical lattice, which will correspond to the single-spin operators $\sigma_{i}^{Z}$ (and $\sigma_{i}^{X}$) in the logical Hamiltonian, remain to be identified. We call these the logical spin operators. We will simply choose one set, the $\sigma_{i}^{Z}$ set, and then attempt to identify the appropriate $\sigma_{i}^{X}$. Remaining consistent with the LHZ construction, we assign our logical *Z* operators to the individual physical spins on the left side diagonal, that is

$$\sigma_{k}^{Z}={\tilde{\sigma }}_{\left(0,k\right)}^{Z}$$ (3)

Clearly, these logical operators commute both with one another and with the stabilizers (all are *z* basis), and they are independent from one another and from the stabilizers (specifying the states of these logical observables would not constrain any of the stabilizer eigenvalues). Therefore, this choice is valid. With this choice, it is of course possible to read the state of logical spins in the *z* basis by measuring physical spins on the left side of the lattice.

We must now identify the logical spin *x* operators with the same properties of commutation and independence, except that the *z* and *x* operators for the same logical spin should anticommute. No set of operators on single physical spins will have this property; instead, we should look for products of physical spin operators. Consider the logical *x* operator for logical spin *i* = 3. Because it must anticommute with the logical operator $\sigma_{Z}^{3}={\tilde{\sigma }}_{\left(0,3\right)}^{Z}$, we include ${\tilde{\sigma }}_{\left(0,3\right)}^{X}$ in the product of operators. However, it should commute with all our earlier stabilizers; thus, it should include zero, two, or four ${\tilde{\sigma }}_{\left(0,3\right)}^{X}$ operators around each plaquette of the lattice (that is, each stabilizer). Finally, it should not include ${\tilde{\sigma }}_{\left(0,k\right)}^{X}$ for any *k* ≠ 3 or else it will not commute with other logical spins. Considering these constraints, we are led to a unique solution: a product of ${\tilde{\sigma }}^{X}$ operators along the path indicated in red in Fig. 1. The other logical *x* operators have analogous forms, descending from the left side and “bouncing” from the base, as shown in the upper left inset in Fig. 1. Formally

$$\sigma_{k}^{X}=\prod_{i}{\tilde{\sigma }}_{\left(i,k\right)}^{X}\prod_{j}{\tilde{\sigma }}_{\left(k,j\right)}^{X}$$ (4)

where *k* = 1, 2, …, *N*, that is, the logical $\sigma_{k}^{X}$ operator is the product of Pauli operators of the set of physical spins with a label of either *i* = *k* or *j* = *k*.

At this point, we are ready to rewrite the original logical spin Hamiltonian in terms of operators on the physical lattice. Each term of the form $h_{j}\sigma_{j}^{Z}$ simply translates to $h_{j}{\tilde{\sigma }}_{\left(0,j\right)}^{Z}$. Meanwhile, each term $J_{i,j}\sigma_{i}^{Z}\sigma_{j}^{Z}$ translates directly to $J_{i,j}{\tilde{\sigma }}_{\left(0,i\right)}^{Z}{\tilde{\sigma }}_{\left(0,j\right)}^{Z}$, but crucially, this can be rewritten as a single physical spin operator $\mu_{i,j}J_{i,j}{\tilde{\sigma }}_{\left(i,j\right)}^{Z}$, where μ_*i*,*j*_ is a certain product of our ν_*i*,*j*_ = ± 1 eigenvalues. To see this, consider first the physical spin at (1,2) and, moreover, the value of the operator ${\tilde{\sigma }}_{\left(1,2\right)}^{Z}$. For all legitimate states, *S*_[0,2]_|*L*〉 = ν_0,2_|*L*〉. However, *S*_[0,2]_ is defined as ${\tilde{\sigma }}_{\left(0,1\right)}^{Z}{\tilde{\sigma }}_{\left(0,2\right)}^{Z}{\tilde{\sigma }}_{\left(1,2\right)}^{Z}$, and the first two of these operators are logical *Z* operators and therefore must be free to take any value; that is, we can make no constraint on ${\tilde{\sigma }}_{\left(0,1\right)}^{Z}|L\rangle$, ${\tilde{\sigma }}_{\left(0,2\right)}^{Z}|L\rangle$ or their product. Then, to ensure that *S*_[0,2]_|*L*〉 = ν_0,2_|*L*〉, we must insist that ${\tilde{\sigma }}_{\left(1,2\right)}^{Z}|L\rangle =\nu_{0,2}{\tilde{\sigma }}_{\left(0,1\right)}^{Z}{\tilde{\sigma }}_{\left(0,2\right)}^{Z}|L\rangle$. Thus, the physical spin at (1,2) is entirely dependent on the physical spins at (0,1) and (0,2), in such a way that the plaquette stabilizer is satisfied. Consequently for the legitimate subspace, we can rewrite the term $J_{1,2}{\tilde{\sigma }}_{\left(0,1\right)}^{Z}{\tilde{\sigma }}_{\left(0,2\right)}^{Z}$ as $\nu_{0,2}J_{1,2}{\tilde{\sigma }}_{\left(1,2\right)}^{Z}$.

Having thus established the dependent nature of the physical spin at (1,2), one can repeat the argument for the physical spin at (1,3): The plaquette stabilizer [0,3] involves two logical spins and the spin whose dependence we have just determined; thus, the dependence of the fourth spin is implied. We find that to ensure *S*_[0,3]_|*L*〉 = ν_0,3_|*L*〉, we will require ${\tilde{\sigma }}_{\left(1,3\right)}^{Z}|L\rangle =\nu_{0,2}\nu_{0,3}{\tilde{\sigma }}_{\left(0,1\right)}^{Z}{\tilde{\sigma }}_{\left(0,3\right)}^{Z}|L\rangle$. One can proceed to establish the dependence of every remaining physical spin; it is always of the form ${\tilde{\sigma }}_{\left(i,j\right)}^{Z}|L\rangle =\mu_{i,j}{\tilde{\sigma }}_{\left(0,i\right)}^{Z}{\tilde{\sigma }}_{\left(0,j\right)}^{Z}|L\rangle$, where μ_*i*,*j*_ is simply a certain product of our chosen stabilizer eigenvalues

$$\mu_{i,j}=\prod_{i^{\prime }=0}^{i-1}\prod_{j^{\prime }=i+1}^{j}\nu_{i^{\prime },j^{\prime }}\;\text{thus},\mu_{i,j}=\pm 1$$ (5)

In general, this is the product of the ν values in a block of the array; in Fig. 1, the green block corresponds to the set of ν values that must be multiplied to determine μ_2,4_.

Now, since we chose to identify ${\tilde{\sigma }}_{\left(0,i\right)}^{Z}$ with the logical *z* operator $\sigma_{i}^{Z}$, we can conclude that each spin (*i*, *j*) encodes the logical product $\sigma_{i}^{Z}\sigma_{j}^{Z}$ (up to the sign μ_*i*,*j*_). Thus, the motivation for our labeling scheme is apparent. We note from the Fig. 1A inset that physical spin (*i*, *j*) also lies at the intersection of the logical *x* operator chains for logical spins *i* and *j*. In a later section, we will show that this is generally the case, for any lattice: If the logical *x* operator for logical spin *i* intersects with a given physical spin, then $\sigma_{i}^{Z}$ is in the product of logical *z* operators to which that spin’s physical *z* operator ${\tilde{\sigma }}_{\left(0,i\right)}^{Z}$ corresponds. This is a more efficient way to identify the roles of physical spins rather than the step-by-step construction described in the previous paragraph.

We can now conclude that the original logical spin system is realized in a subspace of the physical Hamiltonian

$$H_{\text{phys}}=H_{C}+\sum_{j=1}^{N}h_{j}{\tilde{\sigma }}_{\left(0,j\right)}^{Z}+\sum_{i=1}^{N-1}\sum_{j=i+1}^{N}\mu_{i,j}J_{i,j}{\tilde{\sigma }}_{\left(i,j\right)}^{Z}$$ (6)

Here, *H*_C_ encapsulates the physics that forces the physical spins to respect the stabilizer constraints. We see that if we make the choice ν_*i*,*j*_ = +1 for all *i*, *j*, then we recover exactly the same physical prescription proposed by LHZ.

Presently, we also note that this construction allows us to recover the entire spectrum of the logical Hamiltonian, provided that a parity constraint with sufficient strength is introduced. We prove this statement rigorously (and provide bounds on the relevant parameters that guarantee the equivalence) in Materials and Methods.

The constraint Hamiltonian can always be expressed as

$$H_{C}=\sum_{n}E_{n}P_{n}$$ (7)

Here, {*E*_*n*_} are eigenenergies and {*P*_*n*_} are projectors corresponding to eigenstates of *H*_C_. *E*_0_ is the ground energy, and *P*_0_ corresponds to the ground-state subspace of *H*_C_.

If *H*_C_ satisfies the following conditions, logical spins are in the ground state of *H*_logic_ (the logical model) when physical spins are in the ground state of *H*_phys_:

(i) [*P*_0_, *H*_phys_] = 0;

(ii) ∀ [*i*, *j*] : *S*_[*i*,*j*]_*P*_0_ = ν_*i*,*j*_*P*_0_ and $\mu_{i,j}=\prod_{i^{\prime }=0}^{i-1}\prod_{j^{\prime }=i+1}^{j}\nu_{i\prime ,j\prime }$;

(iii) $\forall k,k^{\prime }:[P_{0},\sigma_{k}^{Z}]=[P_{0},U_{k}\sigma_{k}^{X}V_{k}]=[U_{k},\sigma_{k^{\prime }}^{Z}]=[V_{k},\sigma_{k^{\prime }}^{Z}]=0$, where *U*_*k*_ and *V*_*k*_ are unitary operators; and

(iv) $E_{0}+E_{g}<E_{g}^{\prime }$, where *E*_g_ is the ground-state energy of *H*_logic_, $E_{g}^{\prime }$ (*E*_0_ + *E*_g_) is the lowest eigenenergy of *H*_phys_ in the subspace 1 − *P*_0_ (*P*_0_), and 1 is the identity operator.

The proof is given in Materials and Methods. The energy gap between the ground state and the first excited state in the physical model is $e_{\text{phys}}=\;\text{min}\{E_{g}^{\prime }-(E_{0}+E_{g}),e_{\text{logic}}\}$, where *e*_logic_ is the energy gap in the logical model.

Obviously, one way to write down a suitable constraint Hamiltonian is to simply use the stabilizers themselves. For example, if we choose ν_*i*,*j*_ = +1, ∀*i*, *j*, which corresponds to the approach by Lechner *et al*. (*15*), then we can write

$$H_{C}=\frac{\Delta }{2}\sum_{\left[i,j\right]}(1-S_{\left[i,j\right]})$$ (8)

In the ground state of *H*_C_, *E*_0_ = 0 and *S*_[*i*,*j*]_ = + 1;, that is, the ground-state subspace is the subspace encoding *N* logical spins and is 2^*N*^-dimensional. The projector to the ground subspace can be written as

$$P_{0}=\prod_{\left[i,j\right]}\frac{1+S_{\left[i,j\right]}}{2}$$ (9)

Because μ_*i*,*j*_ = +1 (*S*_[*i*,*j*]_*P*_0_ = *P*_0_) and *U*_*k*_ = *V*_*k*_ = 1, conditions (i), (ii), and (iii) are satisfied. When Δ is large enough, condition (iv) can also be satisfied.

However, a Hamiltonian formed by stabilizers involves three- and four-body interactions, which are unphysical. The use of ancilla qutrits to achieve an equivalent but physically realistic *H*_C_ is discussed further in the next section.

Making other choices for the stabilizer values ν_*i*,*j*_ can lead to interesting variants. Consider, for example, the choice ν_*i*,*j*_ = −1 for all *i* and *j*. This means that each local stabilizer requires odd parity among its group of physical spins. Consequently, some of the μ values will be −1 according to Eq. 6. Specifically, μ_*i*,*j*_ = −1 when *i* is odd and *j* is even. The three cases that occur for the *N* = 5 system are marked in Fig. 1B. Thus, these particular *J*_*i*,*j*_ couplings from the original, logical Hamiltonian are multiplied by −1 in the physical Hamiltonian. Presumably, this will not present difficulties for any relevant hardware system because such a system will need to handle both positive and negative *J* values in any case to tackle nontrivial computation problems. However, there is a more profound consequence for the hardware implementation: All our stabilizers now seek to constrain their local groups of spins to odd parities, and this may be easier to realize than the even-parity constraint. Formally, a suitable *H*_C_ can be written in analogous terms to Eq. 9 as $H_{C}=\frac{\Delta }{2}\sum_{\left[i,j\right]}(1+S_{\left[i,j\right]})$, but again, this uses unphysical three- and four-body terms. The interesting distinction is that there is now a natural way of achieving an equivalent *H*_C_ using only a single ancilla qubit for each stabilizer group, as we now discuss.

### Ancillary qubit Ising model

Summarizing the paper so far, the previous section introduced a stabilizer formalism and used it to map a Hamiltonian with *N* logical spins having “all-to-all” interactions to a physical Hamiltonian with *N*(*N* + 1)/2 physical spins but requiring only local interactions. The nature of the local stabilizer rules was defined by our choice of constants ν_*i*,*j*_. We noted that the choice of setting ν_*i*,*j*_ = +1 for all *i*, *j* results in the prescription given in LHZ; that is, the local constraints on groups of four or three spins are equivalent to demanding even parity in the number of spins aligned to the negative *z* direction. The next most natural choice is ν_*i*,*j*_ = −1 for all *i*, *j*. This leads to some μ_*i*,*j*_ = −1 factors in the physical Hamiltonian, but moreover, it inverts the parity requirements on all local groups from even to odd. The distinction between even- and odd-parity constraints seems relatively minor when the constraining Hamiltonian *H*_C_ is written formally using the stabilizers, as in Eq. 9. However, because the stabilizers are three- and four-body terms, this does not suffice as a physical prescription, and instead, one must find a realizable *H*_C_ that is equivalent.

For the even-parity case, LHZ suggested the introduction of an ancilla qutrit, that is, a spin-1 system, for each group of physical spins (they remark that the role can be played equivalently with qubits rather than qutrits). Following their prescription, we can write

$$H_{C}^{\text{even}}=\frac{\Delta }{4}\sum_{\left[i,j\right]}H_{\left[i,j\right]}$$

where

$$H_{\left[i,j\right]}=\{\begin{array}{cc} {\left(4T_{\left[i,j\right]}^{Z}+\sum_{a}{\tilde{\sigma }}_{a}^{Z}\right)}^{2} & \text{if}\;i+2<j\\ {\left(1+4T_{\left[i,j\right]}^{Z}+\sum_{a}{\tilde{\sigma }}_{a}^{Z}\right)}^{2} & \text{if}\;i+2=j \end{array}$$ (10)

where $T_{\left[i,j\right]}^{Z}$ is the spin-1 (qutrit) ancilla associated with lattice plaquette [*i*, *j*], with eigenvalues −1, 0, and +1. The sums run over the cases (*i*, *j*), (*i*, *j* − 1), (*i* + 1, *j*), and (*i* + 1, *j* − 1) or just the first three for the *i* + 2 = *j* instances. For these latter instances, as an alternative to giving them a distinct *H*_[*i*,*j*]_, it is possible to instead introduce “dummy” physical spin-^1^/_2_ systems that form an additional row beneath but which are “locked” to the ${\tilde{\sigma }}_{a}^{Z}=+1$ eigenstate; the physics is identical.

The degenerate ground state of $H_{C}^{\text{even}}$ is a subspace formed from all the “correct” even-parity configurations of physical spins where each is matched with a correlated state of the ancilla spin. Note that the ancilla has no role in *H*_phys_ outside of $H_{C}^{\text{even}}$. An intuition behind the use of the ancilla is as follows: Because the term is squared, the lowest energy contribution it can make is 0. Note that the sum of the four ${\tilde{\sigma }}^{Z}$ operators can take values equal to −4, −2, 0, 2, or 4, and the value of the 4*T*^Z^ operator can be equal to −4, 0, or 4. Therefore, if the physical spins sum to ± 2, there is no assignment of the qutrit that can achieve a total energy of zero, but −4, 0, and 4 are acceptable. These are of course precisely the even-parity states.

Now, we consider the equivalent cases for our “always odd-parity” scenario, which we obtained by considering the ν_*i*,*j*_ = − 1 case. Again, we must identify a simple physical *H*_C_ with a correct degenerate ground state. As with the LHZ example, we again use the technique of squaring a sum of $\sum_{a}{\tilde{\sigma }}_{a}^{Z}$ operators, but now, we find that the ancilla needs to take only two values. Specifically, we opt for

$$H_{C}^{\text{odd}}=\frac{\Delta }{4}\sum_{\left[i,j\right]}H_{\left[i,j\right]}$$

where

$$H_{\left[i,j\right]}=\{\begin{array}{cc} {\left(2{\tilde{\sigma }}_{\left[i,j\right]}^{Z}+\sum_{a}{\tilde{\sigma }}_{a}^{Z}\right)}^{2} & \text{if}\;i+2<j\\ {\left(1+2{\tilde{\sigma }}_{\left[i,j\right]}^{Z}+\sum_{a}{\tilde{\sigma }}_{a}^{Z}\right)}^{2} & \text{if}\;i+2=j \end{array}$$ (11)

Here, ${\tilde{\sigma }}_{\left[i,j\right]}^{Z}$ (note the square brackets [ ] in the subscript) is the spin-^1^/_2_ ancilla associated with lattice plaquette [*i*, *j*]. As before, the sums run over the cases (*i*, *j*), (*i*, *j* − 1), (*i* + 1, *j*), and (*i* + 1, *j* − 1) or just the first three for the *i* + 2 = *j* instances. Again, one could introduce a row of dummy physical spins below the active array to make all plaquettes of the array square so that the *i* + 2 = *j* cases are no longer special. This follows the form of the LHZ construct exactly, except for the $2{\tilde{\sigma }}_{\left[i,j\right]}^{Z}$ term, which, of course, has values ± 2. The same intuition just described therefore leads us to see that the ground state will be spanned by states where the $\sum_{a}{\tilde{\sigma }}_{a}^{Z}$ yields ± 2, that is, the odd-parity states. In Materials and Methods, we show that such a constraint Hamiltonian satisfies conditions (i) to (iv) if Δ is large enough.

The required interactions are encouragingly simple. Assuming that the physical implementation uses row dummy spins, then expanding the squared expression in Eq. 14 and neglecting global shifts gives

$$H_{\left[i,j\right]}=\sum_{a\neq b}{\tilde{\sigma }}_{a}^{Z}{\tilde{\sigma }}_{b}^{Z}+2\sum_{a}{\tilde{\sigma }}_{a}^{Z}{\tilde{\sigma }}_{\left[i,j\right]}^{Z}$$ (12)

where the *a* and *b* indices here run over the local physical spins as usual. This appears to be a potential advantage over the even-parity solution with its qutrit (spin-1) ancilla, not only because qubit (spin-^1^/_2_) systems may be easier to realize but also because the expansion of Eq. 12 has terms (*T*^Z^)^2^, that may be awkward to realize; for the odd-parity version, the equivalent terms ${\left({\tilde{\sigma }}_{\left[i,j\right]}^{Z}\right)}^{2}$ are merely the identity and can be neglected.

Equation 15 involves interactions between all four spins defining the lattice plaquette and an interaction of the same form but double the strength between each of these and their shared ancilla spin. The ratio of 2 between these strengths is not required; it is optimal, but any ratio greater than 1 but less than 3 will correctly reproduce the effect of the stabilizers. It is interesting to speculate that this set of interactions might be very naturally realized by connecting the four physical spins to a common resonator and coupling this group’s ancilla to the same resonator with twice the coupling strength. This is indicated schematically in Fig. 1C.

We note that very recently, two new papers (*20*, *21*) that discuss superconducting systems capable of supporting *M*-body parity constraints (stabilizers, in our language) have been posted online. Another study (*22*) has shown how these *M*-body parity constraints can be implemented on the Chimera graph; it would be interesting to contrast implementations on that established platform with the new, dedicated stabilizer-based architectures we envisage here.

### Spectrum and numerical results

The preceding sections provide an analytic treatment within which both the LHZ proposal and a variant proposal based on odd parity have emerged as examples of local Hamiltonians that can simulate all-to-all interactions. Before moving on to considering new forms of physical spin layout, we wish to compare these two alternatives using a numerical study of small systems.

Generally, the analytic conclusions described earlier are valid when the energy Δ associated with the parity-constraining terms is sufficiently large compared to other terms. It is interesting to see how these two approaches perform for finite values of the parameters.

We performed our simulations using exact diagonalization. Because the number of ancillas required is (*N* − 1)*N*/2, the total number of physical spins required is (*N* + 1)*N*/2 + *N*(*N* − 1)/2 = *N*^2^ (if we consider triangular constraints in the bottom layer). This quadratic scaling severely limits our ability to simulate even small systems numerically, especially when using ancillary qutrits (see Table 1).

**Table 1:** Scaling of the computational space. The size of the computational space scales quadratically with the number of logical spins *N*. Left column, size of the logical system; middle column, size of the computational space for the ancillary qutrit (even parity) architecture; right column, size of the computational space for the ancillary qubit (odd-parity) architecture.

***N***	**Qutrit**	**Qubit**
3	2^6^3^3^	2^9^
4	2^10^3^6^	2^16^
5	2^15^3^10^	2^25^
6	2^21^3^15^	2^36^
7	2^28^3^21^	2^49^

As discussed by Lechner *et al*. (*15*), the strength of the constraint terms Δ is one of the key adjustable parameters of the architecture. The analytic arguments in the preceding section rely on Δ being the dominant energy at the end of the adiabatic sweep such that the correct stabilizers are enforced. On the other hand, we also wish for the energy scales (*h* and *J*) to be as large as possible because their magnitude will influence the gap between the ground state of the logical Hamiltonian and its excited states and thus determine the speed and practicality of QA or other adiabatic processes. Consequently, it is interesting to see how close we can permit those lesser energy scales to come to Δ or, in other words, how modest a ratio will suffice.

Presently, we note that there is another reason to be interested in the modest values of this ratio: the detectability and correctability of errors in the system’s evolution. In our simulations, we benchmark the different parity-enforcing terms with two different metrics while varying the strength of Δ. We use a random Ising model where the *J*_*ij*_ elements are drawn from a uniform distribution in [−*J*, *J*]. The energies *h*_*i*_ are drawn from the same distribution. The quantity *J*_av_ is the average unsigned value, that is, *J*/2. We will be interested in the ratio *R* between Δ, the energy scale of the parity-constraining terms, and *J*_av_.

**Fig. 2:**
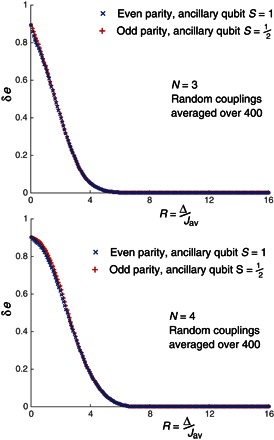
Energy levels at the end of the adiabatic sweep. Deviation between the lowest energy gap in the logical Hamiltonian ***H*_logic_** and the physical Hamiltonian ***H*_phys_** as a function of the constraint’s strength. These graphs demonstrate that the even- and odd-parity variants perform almost identically.

Following along the same lines as the numerical analysis in Lechner *et al*. (*15*), for our first metric, we take the system to be at the end of its adiabatic sweep, and we find the gap between the ground state and lowest excited state(s). We find this gap for the physical system, that is, the *N*(*N* + 1)/2 array of physical spins, and see how it deviates from the same quantity found using the ideal logical Hamiltonian. That is to say, we plot

$$\delta e=|e_{\text{logic}}-e_{\text{phys}}|$$

where *e*_logic_ = λ_logic_(1) − λ_logic_(0), *e*_phys_ = λ_phys_(1) − λ_phys_(0), and λ(*i*) is the *i*th eigenvalue of a given architecture. Locating the value of Δ where this deviation largely vanishes gives insight into how large Δ should be for the mapping process to be successful. Figure 2 shows the results for systems of *N* = 3 and *N* = 4 logical spins (that is, 9 and 16 physical spins, respectively). The behavior is as expected; there is no significant difference between the even-parity physical architecture (with its spin-1 qutrit ancillas) and the odd-parity system (using spin-^1^/_2_ qubit ancillas).

Perhaps the more crucial energy in a QA process is not the gap at the end of the anneal but rather the minimum gap that occurs at any time throughout the evolution. It is this gap that is usually used to characterize the stability of the process and the speed with which it can be completed. Our second metric concerns this minimum gap as we sweep between an initial Hamiltonian $\sum_{i}{\tilde{\sigma }}_{i}^{X}$ (where the sum runs over all the physical spins) and the final form. We plot the ratio χ of the minimum gap occurring in the physical architecture (which depends on *R*) to that which would occur in the logical system

$$\chi (R)={\text{MinGap}}_{\text{phys}}/{\text{MinGap}}_{\text{logic}}$$

**Fig. 3:**
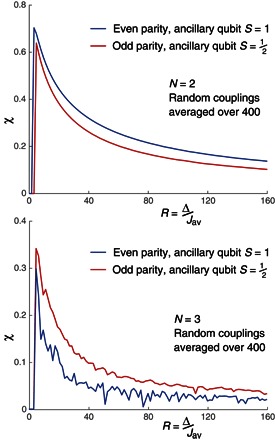
Scaling of the minimum gap. Minimum gap ratio between the physical and logical systems as a function of the constraint’s strength. The even- and odd-parity variants perform differently, especially in the ***N* = 3** case, as discussed in the text.

In Fig. 3, we show the behavior of χ for the smallest two nontrivial systems: *N* = 2 and *N* = 3 logical spins. Each data point is an average of 400 simulations, and we have chosen to find the average of $\frac{1}{\chi }$ and then reciprocate; this emphasizes cases where the gap in the physical system vanishes (or nearly vanishes). We do see some variation between the behavior of the even-parity–constraining system with its spin-1 ancillas and the alternative odd-parity architecture using spin-^1^/_2_ ancillas. The curve of the former approaches the *x* axis many times, suggesting level crossing for the ancillary qutrit implementation that is not present when using the ancillary qubit version.

### Remarks on error detection and correction

The analytics portion of this paper stresses the significance of the stabilizer-enforcing terms and may lead to the supposition that we would like to have Δ ≫ *J*, *h*. However, from our small system numerics, we note that it can suffice for these energy ratios to be modest, and from Fig. 3, we see that larger values of this ratio can be associated with smaller values of the gap. This may lead to speculation that moderately large values of the ratio *R* = Δ/*J*_av_ are optimal, and the following speculative line of thought leads to the same conclusion.

As pointed out by Pastawski and Preskill (*23*), the multiple parity constraints applied to the physical system have the consequence that, if the final state is read out incorrectly, it is highly likely that classical postprocessing can recover the correct set of measurements. The threshold for error correction, that is, the number of spins that would need to be misread before the correct state cannot be inferred, is very high. Can this permit us to correct an error that occurs during the evolution, that is, a jump from the ground state to an excited state? This depends on whether the excited state in question has different parity values. If *R* is very large compared to other energies, then the spectrum will be such that that the low-lying excited states have correct parity, and therefore jumps to these states are uncorrectable (and undetectable, except that the final measured state constitutes a poor solution to the optimization problem). However, opting for a more modest *R* value may permit the low-lying excited states to violate the parity constraints and thus conceivably permit us to correct them after measurement. To explore this point further, suppose that a system is configured such that the constraints Δ will achieve the highest value possible at the end of the anneal, given the physical nature of the hardware; further, suppose that the strengths of the *J* and *h* terms can be freely adjusted. We hypothesize that, ideally, their strength should be some significant portion of Δ, so that *R* is only modest. In this way, we would hope to overlap the spectrum of the two parity subspaces without increasing the possibility of having an error. Exploring the validity of this principle would be an interesting challenge for future work.

### New layouts supporting arbitrary connectivity

So far, we have used our stabilizer formalism only to recover the proposal of LHZ, but including the freedom to choose odd- versus even-parity constraints. However, the stabilizer picture can allow us to design a wide range of physical layouts to realize different levels of connectivity and/or higher-order correlations compared to the two-body case.

**Fig. 4:**
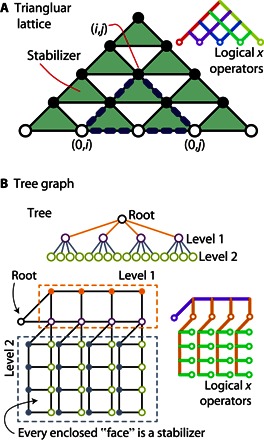
Lattice variants. (**A**) The lattice for encoding all-to-all connected logical spins using three-spin stabilizers. Here, the number of physical spins is ***N*_*P*_ = 15** and the number of logical spins is ***N* = 5**. The number of stabilizers must then be ***N*_S_** = ***N***_P_ − ***N* = 10**, and we choose these to be triangular stabilizers (green triangles). Each circle denotes a physical spin. Empty circles are vertex spins **(0, *j*)**, and solid circles are edge spins **(*i* ≠ 0,** ***j*)**. Spins **(0,** ***i*)**, **(0,** ***j*)**, and **(*i***, ***j*)** always form an isosceles triangle (marked with the dashed blue line). (**B**) Stabilizer code of the tree graph logical model. The top figure depicts the logical model, the bottom left figure depicts the physical lattice used to encode the logical model, and the bottom right figure depicts the chains of logical *x* operators. Each empty circle denotes a vertex physical spin (or a logical spin in the tree graph), and each solid circle denotes an edge spin corresponding to the two-body Ising interaction between two logical spins. Each plaquette corresponds to a stabilizer, as in Fig. 1A.

Before varying the nature of the logical Hamiltonian, we note that our approach can guide us to layouts that support exactly the same logical Hamiltonian as the LHZ construction but which have different kinds of stabilizers (rather than merely different stabilizer signs, as considered earlier). Perhaps the simplest set of stabilizers is the triangular pattern shown in Fig. 4A. When finding sets of stabilizers such as these, it is helpful to remember the principle that any product of stabilizers is also a stabilizer. This can allow one to translate from stabilizers that are nonlocal in the physical layout to a local set. For example, the product of the three stabilizers within the large blue dashed triangle is equivalent to the stabilizer ${\tilde{\sigma }}_{\left(0,i\right)}^{Z}{\tilde{\sigma }}_{\left(0,j\right)}^{Z}{\tilde{\sigma }}_{\left(i,j\right)}^{Z}$, which involves only the spins at the corners of that triangle (because each spin in the middle of an edge appears twice in the product, the spins canceled out). This layout, or others generated using the same principles, may prove to be more natural to implement with a given technology.

We now proceed to introduce the formalism that will allow us to embed Hamiltonians with bespoke connectivity. For the moment, we will continue to restrict our logical Hamiltonians to involve only one- and two-body terms. We therefore consider a connectivity graph with vertices *V*, representing the logical spins, and edges *E*, representing the required terms in the logical Hamiltonian (Eq. 1). That is to say, if *J*_*i*,*j*_ ≠ 0, then the edge linking vertex *i* and vertex *j* are present in set *E*. We will need |*V*| + |*E*| physical spins if we wish to encode |*V*| = *N* logical spins, that is, one physical spin (0, *i*) for each vertex *i* ∈ *V* and one physical spin (*i*, *j*) for each edge (*i*, *j*) ∈ *E*.

We only need to follow our earlier prescription: We select *N* of the physical spins to represent the logical *z* operators; that is, we identify the physical spins (0, *i*) for which ${\tilde{\sigma }}_{0,i}^{Z}$ is identified with $\sigma_{i}^{Z}$. We then define |*E*| independent stabilizers, as before, specifying each stabilizer as a product of ${\tilde{\sigma }}^{Z}$ operators. Finally, we determine the logical *x* operators that are implied by these choices, recalling the following requirements: operator $\sigma_{i}^{X}$ must commute with all other logical *x* operators and with all the stabilizers, and it must commute with all $\sigma_{j\neq i}^{Z}$ while anticommuting with $\sigma_{i}^{Z}$. As before, this leads us to the rule that the product of physical ${\tilde{\sigma }}^{X}$ operators, which constitutes a given logical *x* operator $\sigma_{i}^{X}$, must (i) include ${\tilde{\sigma }}_{i}^{X}$, (ii) exclude all ${\tilde{\sigma }}_{j\neq j}^{X}$, and (iii) include an even number (or zero) of operators that address spins in each stabilizer.

Assuming that all logical *x* operators have been identified, we can now identify the roles of the remaining physical spins using the following rule: The physical spin where logical *x* operators $\sigma_{i}^{X}$ and $\sigma_{j}^{X}$ intersect is to be labeled (*i*, *j*). This spin’s physical *z* operator ${\tilde{\sigma }}_{i,j}^{Z}$ is identical to the logical two-body term $\sigma_{i}^{Z}\sigma_{j}^{Z}$, up to a sign μ_*i*,*j*_. The sign is simply a function of which stabilizers we have chosen to be negative, as in the example leading to Eq. 6. In an earlier section, we alluded to this convenient rule, and we now justify it.

Together, the logical σ^Z^ operators and the stabilizers form a total of |*V*| + |*E*| independent operators, each of which is either a physical operator ${\tilde{\sigma }}^{Z}$ or a product of such operators. From their independence, it follows that we must be able to express any operator ${\tilde{\sigma }}_{\left(i,j\right)}^{Z}$ as a product of logical operators and stabilizers, that is

$${\tilde{\sigma }}_{\left(i,j\right)}^{Z}=(\text{product of}\;\sigma^{Z})\times (\text{product of stabilizers})$$

We can determine which logical σ^Z^ operators are in this product by considering logical σ^X^ operators. If $\sigma_{k}^{Z}$ is in the product, ${\tilde{\sigma }}_{\left(i,j\right)}^{Z}$ anticommutes with $\sigma_{k}^{X}$; otherwise, ${\tilde{\sigma }}_{\left(i,j\right)}^{Z}$ commutes with $\sigma_{k}^{X}$. However, from our definition of the logical *x* operators, only $\sigma_{i}^{X}$ and $\sigma_{j}^{X}$ anticommute with ${\tilde{\sigma }}_{\left(i,j\right)}^{Z}$; thus, we conclude that only $\sigma_{i}^{Z}$ and $\sigma_{j}^{Z}$ are in the product, that is

$${\tilde{\sigma }}_{\left(i,j\right)}^{Z}=\sigma_{i}^{Z}\sigma_{j}^{Z}\times (\text{product of stabilizers})$$

Therefore, the Ising interaction $J_{i,j}\sigma_{i}^{Z}\sigma_{j}^{Z}$ in the logical model can be mapped to $J_{i,j}{\tilde{\sigma }}_{i,j}^{Z}$ in the physical model (up to a sign determined by the value of stabilizers).

In practice, this means that if we wish to have a physical spin representing a two-body term $\sigma_{i}^{Z}\sigma_{j}^{Z}$ in the logical Hamiltonian, that is, if that edge exists in *E*, then the lines of physical spins associated with the two logical *x* operators must cross. This provides a design principle to create a bespoke physical array to represent a given logical Hamiltonian. We can see that the square lattice of LHZ in Fig. 1 and the triangular lattice in Fig. 4A meet this condition for a fully connected graph; that is, intersects with every other logical *x* operator, and so all edges exist. However, the layout in Fig. 4B supports a more restricted graph, that is, a three-tier hierarchical tree, and consequently requires only 2*N* − 1 physical spins for *N* logical variables. For a logical Hamiltonian with exactly this connectivity, this bespoke layout therefore provides a more efficient representation, and presumably, the gap during annealing may be larger.

**Fig. 5:**
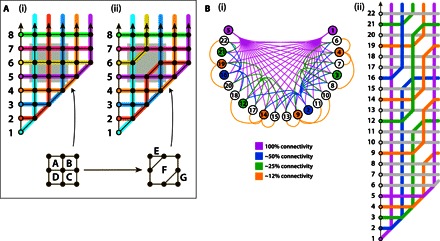
Lattices with bespoke connectivity. (**A**) Strategic disruptions to the stabilizer lattice can control the routing of logical *x* operators. Starting from a regular lattice section (i) and replacing the central four stabilizers A, B, C, and D with three stabilizers E, F, and G (where F is a six-body stabilizer) and removing a physical spin results in a new lattice (ii) where the paths of the *x* operators for logical spins 2 and 6 are reflected. (**B**) Designing nontrivial connectivities via such reflections: Diagram (i) is a connectivity graph showing a highly connected (but not all-to-all) relationship between 22 nodes. Diagram (ii) is a lattice formed from three-, four-, and six-body stabilizers, arranged so as to realize that connectivity; for every linked pair in (i), there is at least one physical spin in (ii) representing the relative orientation of the logical spins.

Figure 5 (A and B) provides further examples of interesting bespoke layouts. The layout in Fig. 5A(i) is a simple pattern with a logical connectivity graph such that vertices (that is, logical spins) 1 to 5 are connected to all other vertices, whereas vertex 6 and higher do not interconnect among themselves. An interesting variant occurs if we remove one physical spin and correspondingly reduce the stabilizer count by one, as shown in Fig. 5A(ii). Note that a central group of four square stabilizers has been replaced with two triangular stabilizers and a single hexagonal stabilizer. [As an aside, we note that a suitable six-body, negative-parity stabilizer can be realized using only two ancilla qubits (see Materials and Methods).] The effect of this central disruption to the layout is that it effectively “reflects” the logical *x* operator chains that would have passed through it. This alters the logical connectivity graph (for example, logical spin number 2 now only connects to the logical spins numbered 1, 3, 4, and 5). There are now two physical spins for each of the labels ${\tilde{\sigma }}_{2,3}^{Z}$, ${\tilde{\sigma }}_{2,4}^{Z}$, and ${\tilde{\sigma }}_{2,5}^{Z}$. This does not present an in-principle difficulty when translating from the logical Hamiltonian to the local fields on the physical spins; we simply need to ensure that the total field on the two spins labeled ${\tilde{\sigma }}_{2,3}^{Z}$ is equal to the factor *J*_2,3_ in the logical Hamiltonian and similarly for the other local fields.

The “reflecting” stabilizer in Fig. 5A(ii) can be used repeatedly within a larger lattice to control the interactions between logical *x* operators and thus define the logical connectivity graph. This is illustrated in Fig. 5B. Here, the layout realizes a rather complex connectivity graph in which there are a small number of highly connected vertices, a larger number of more modestly connected nodes, and so on. One might, for example, choose the width of the strip of physical spins to be log (*N*) while its length is *N*, where *N* is the number of logical spins. With these *N* log (*N*) spins, one could engineer a hierarchy where one logical spin connects to all others, two connect to 50% of the set, four connect to 25%, and so on.

### New layouts supporting higher-order interactions

**Fig. 6:**
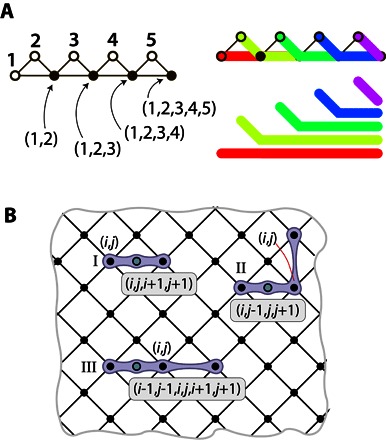
Lattices with higher-order interactions. (**A** and **B**) Methods for representing multibody interactions. In (A), this is achieved by a ladder of stabilizers; on the left figure, filled circles represent increasingly high-order correlations. The top right figure shows how the chains of logical *x* operators intersect, whereas the bottom right figure shows, for clarity, the same chains but without overlapping. In (B), the lattice notation follows that of Fig. 1A. Green circles denote additional physical spins representing multibody interactions. Purple links denote stabilizers. In case I, the stabilizer of physical spins **(*i*, *j*)** and **(*i* + 1, *j* + 1)** and of the additional physical spin corresponds to the four-body term $\sigma_{i}^{Z}LZj\sigma_{i+1}^{Z}\sigma_{j+1}^{Z}$; in case II, the stabilizer of physical spins **(*i* − 1, *j* − 1)**, **(*i***, ***j*)**, and **(*i* − 1, *j* + 1)** and of the additional physical spin corresponds to the four-body term $\sigma_{i}^{Z}\sigma_{j-1}^{Z}\sigma_{j}^{Z}\sigma_{j+1}^{Z}$; and in case III, the stabilizer of physical spins **(*i* − 1, *j* − 1)**, **(*i*, *j*)**, and **(*i* + 1, *j* + 1)** and of the additional physical spin corresponds to the six-body term $\sigma_{i-1}^{Z}\sigma_{j-1}^{Z}\sigma_{i}^{Z}\sigma_{j}^{Z}\sigma_{i+1}^{Z}\sigma_{j+1}^{Z}$.

Finally, we consider how the stabilizer picture presented here generalizes to support terms in the logical Hamiltonian that are higher than two-body terms. Previously, we noted that when two logical *x* operators *i* and *j* intersect, the physical spin at the intersection necessarily represents the two-body logical operator $\sigma_{i}^{Z}\sigma_{j}^{Z}$. However, it is possible for multiple logical operators to intersect at a specific physical spin, as shown in Fig. 6A. Then, the same arguments developed above apply, so that when the logical *x* operators for logical spins *i*, *j*,…, *p* all intersect, then the physical operator ${\tilde{\sigma }}^{Z}$ on that spin will correspond to the logical product $\sigma_{i}^{Z}\sigma_{j}^{Z}\ldots \sigma_{p}^{Z}$, up to a sign determined by the use of negative stabilizer constraints.

Figure 6B provides a second illustration of how higher-order terms can be introduced. The figure shows a region of the standard LHZ layout, that is, a larger version of the pattern shown in Fig. 1. There are three additional physical spins (green circles) and, correspondingly, three additional stabilizers (gray-shaded regions). Each of the new physical spins provides a high-order logical correlation, as specified in the caption.

Note that the ideas presented here could potentially be used in addition to the principle of minor embedding rather than replacing that approach outright. Starting from the original logical Hamiltonian, which directly corresponds to the structure of some computational task, one might use the minor embedding principle to derive a second, intermediate logical Hamiltonian with a larger number of spins. This intermediate Hamiltonian could then be translated into a stabilizer-based layout as described here. The potential benefit would be an increased flexibility in the connectivity offered by a given layout pattern.

## DISCUSSION

We began by presenting a stabilizer formulation for the problem of mapping a Hamiltonian with *N* all-to-all interacting spins to a Hamiltonian of *N*(*N* + 1)/2 spins with only local interactions. As a first illustration of the approach, we took the recent work of Lechner *et al*. (*15*) and adopted their physical spin layout. We noted the resulting logical *z* and logical *x* operators; the latter are chains of operators that traverse the lattice (as occurs in topological error-correcting codes). We recovered the LHZ result, and we also identified an interesting variant based on constraining local groups of spins to odd parity, rather than to even parity. This variant has an attractively simple realization in terms of pure Ising interactions and ancilla qubits (rather than qutrits) and might perhaps be realized through commonly shared resonators. We numerically verified our results for small systems of *N* = 2, 3, 4 logical spins. There are some indications that our new odd-parity, qubit-ancilla model may maintain a more reliable energy gap during an anneal.

Having thus demonstrated our formalism in an established context, we proceed to show how it can be used for a wide range of different physical spin layouts. We display a triangle lattice for all-to-all connectivity, before moving on to create layouts, which support specific (less than all-to-all) connectivities with the advantage that fewer than Order (*N*^2^) physical spins are needed. Our examples include a three-tier tree structure requiring 2*N* − 1 physical spins and a more complex pattern offering a range of connectivities with *N* log (*N*) physical spins. Finally, we show that there is no constraint to two-body logical terms; even within a strictly 2D layout, arbitrarily high-order logical terms can be realized in a natural way.

## MATERIALS AND METHODS

### Sufficient condition of the parity constraint

Because [*P*_0_, *H*_phys_] = 0, *P*_0_ is a subspace of *H*_phys_, ; that is, *H*_phys_ can be rewritten as *H*_phys_ = *P*_0_*H*_phys_*P*_0_ + (1 − *P*_0_)*H*_phys_(1 − *P*_0_). In the subspace *P*_0_, the spectrum is determined by the effective Hamiltonian *H*_eff_ = *P*_0_*H*_phys_*P*_0_ = *H*_phys_*P*_0_.

Using stabilizers, single-spin Pauli operators can be expressed as

$${\tilde{\sigma }}_{\left(i\neq 0,j\right)}^{Z}={\tilde{\sigma }}_{\left(0,i\right)}^{Z}{\tilde{\sigma }}_{\left(0,j\right)}^{Z}\prod_{i^{\prime }=0}^{i-1}\prod_{j^{\prime }=i+1}^{j}S_{\left[i^{\prime },j^{\prime }\right]}$$ (13)

where the product of stabilizers corresponds to a rectangular area (with a corner cut) composed of plaquettes in the lattice (see Fig. 1): The bottom plaquette is a triangle plaquette rather than a square, the top left side of the area connects spins (0, *i*) and (0, *j*), and the top right side of the area connects spins (*i*, 0) and (0, *j*). As an example, the area corresponding to ${\tilde{\sigma }}_{\left(i\neq 2,4\right)}^{Z}$ is highlighted in green in Fig. 1. One can find that, in the product of stabilizers, each Pauli operator occurs for even times, except ${\tilde{\sigma }}_{\left(0,i\right)}^{Z}$, ${\tilde{\sigma }}_{\left(0,j\right)}^{Z}$, and ${\tilde{\sigma }}_{\left(i,j\right)}^{Z}$. Using Eq. 16 and condition (ii), we have

$$H_{\text{eff}}=H_{\text{phys}}P_{0}=E_{0}P_{0}+H_{\text{logic}}P_{0}$$ (14)

*P*_0_ can be written as

$$P_{0}=\sum_{\alpha }P_{0,\alpha }$$ (15)

where α = (α_1_, α_2_, …, α_*N*_) is a string of α_*k*_ = ±1, and $P_{0,\alpha }=P_{0}{\bar{P}}_{\alpha }$, with

$${\bar{P}}_{\alpha }=\prod_{k}\frac{1+\alpha_{k}\sigma_{k}^{Z}}{2}$$ (16)

being the projector to the subspace where logical spin operators $\left\{\sigma_{k}^{Z}\right\}$ take eigenvalues {α_*k*_}. Because $[P_{0},\sigma_{k}^{Z}]=0$, {*P*_0,α_} are projectors; that is, $P_{0,\alpha }^{2}=P_{0,\alpha }$.

For any two sets of eigenvalues α and α′, we introduce a unitary operator

$$U_{\alpha ,\alpha^{\prime }}=\prod_{k}[\delta_{\alpha_{k},\alpha_{k}^{\prime }}1+(1-\delta_{\alpha_{k},\alpha_{k}^{\prime }})U_{k}\sigma_{k}^{X}V_{k}]$$ (17)

Then

$$U_{\alpha ,\alpha^{\prime }}P_{0,\alpha^{\prime }}U_{\alpha ,\alpha^{\prime }}^{†}=P_{0,\alpha }$$ (18)

Here, we have used condition (iii). Therefore, dimensions of subspaces {*P*_0,α_} are the same, and the dimension *D* = Tr(*P*_0,α_) = Tr(*P*_0_)/2^*N*^.

Common eigenstates of $\left\{\sigma_{k}^{Z}\right\}$ are eigenstates of *H*_logic_, and the eigenvalue only depends on α, that is

$$H_{\text{logic}}{\bar{P}}_{\alpha }={\bar{E}}_{\alpha }{\bar{P}}_{\alpha }$$ (19)

where

$${\bar{E}}_{\alpha }=\sum_{i=1}^{N}h_{i}\alpha_{i}+\sum_{i=1}^{N-1}\sum_{j=i+1}^{N}J_{i,j}\alpha_{i}\alpha_{j}$$ (20)

Then, the effective Hamiltonian can be rewritten in the diagonalized form

$$H_{\text{eff}}=\sum_{\alpha }(E_{0}+{\bar{E}}_{\alpha })P_{0,\alpha }$$ (21)

Here, *E*_0_ is the ground-state energy of the constraint Hamiltonian *H*_C_, ${\bar{E}}_{\alpha }$ is the energy of the logical-spin configuration α in the logical Hamiltonian *H*_logic_ and *P*_0,α_ is the projector to the corresponding eigenstate subspace. Therefore, we see that, in the subspace *P*_0_, the physical Hamiltonian *H*_phys_ and the logical Hamiltonian *H*_logic_ have the same spectrum. We would like to remark that not only the spectrum but also the eigenstates of the two Hamiltonians coincide with each other in the subspace *P*_0_, ; that is, the same eigenenergy ${\bar{E}}_{\alpha }$ corresponds to the same configuration α of logical spins.

The whole spectrum of *H*_phys_ is composed of the spectrum in the subspace *P*_0_ (the spectrum of *H*_eff_) and the spectrum in other subspaces [the spectrum of (1 − *P*_0_)*H*_phys_(1 − *P*_0_)]. Here, the ground-state energy of *H*_eff_ is $E_{0,g}=E_{0}+\text{min}\{{\bar{E}}_{\alpha }\}=E_{0}+E_{g}$ and the ground-state energy of (1 − *P*_0_)*H*_phys_(1 − *P*_0_) is $E_{g}^{\prime }$. Therefore, when $E_{0}+E_{g}<E_{g}^{\prime }$, *E*_0,g_ is the ground-state energy of the whole spectrum; that is, the ground state of *H*_phys_ is the ground state of *H*_eff_. We note that in the ground state of *H*_eff_, the logical spins are in the ground state of *H*_logic_.

Assuming that *E*_1_ is the first excited state energy of the constraint Hamiltonian *H*_C_, the ground state energy of the (1 − *P*_0_)*H*_phys_(1 − *P*_0_) has a lower bound $E_{g}^{\prime }\geq E_{1}-E_{\text{max}}$, where $E_{\text{max}}=\sum_{i=1}^{N}|h_{i}|+\sum_{i=1}^{N-1}\sum_{j=i+1}^{N}|J_{i,j}|$. Because $\sum_{\alpha }{\bar{E}}_{\alpha }=0$, *E*_g_ has an upper bound *E*_g_ ≤ 0. Therefore, when *E*_1_ − *E*_0_ > *E*_max_, the condition $E_{0}+E_{g}<E_{g}^{\prime }$ is satisfied, and the ground state of *H*_phys_ corresponds to the ground state of *H*_logic_. Finally, because *E*_max_ is the upper bound of ${\bar{E}}_{\alpha }$, when *E*_1_ − *E*_0_ > 2*E*_max_, $E_{g}^{\prime }$ is higher than the energy of any state in the subspace *P*_0_, and the whole spectrum of *H*_logic_ is reproduced by low-lying states of *H*_phys_.

### Group subspace of the ancillary qubit model

We define *M*_[*i*,*j*]_ as the number of excitations (number of spins along the −*z* direction), and

$$M_{\left[i,j\right]}=\{\begin{array}{cc} 21-\frac{1}{2}\sum_{a}{\tilde{\sigma }}_{a}^{Z} & \text{if}\;i+2<j\\ \frac{3}{2}1-\frac{1}{2}\sum_{a}{\tilde{\sigma }}_{a}^{Z} & \text{if}\;i+2=j \end{array}$$ (22)

Here, the sums run over the cases (*i*, *j*), (*i*, *j* − 1), (*i* + 1, *j*), and (*i* + 1, *j* − 1) or just the first three for the *i* + 2 = *j* instances. Thus, in the ground state of *H*_C_, *E*_0_ = 0, *M*_[*i*,*j*]_ = 1 or 3, and *S*_[*i*,*j*]_ = −1 for all stabilizers.

The projector to the ground state subspace is

$$P_{0}=\prod_{\left[i,j\right]}P_{\left[i,j\right]}$$ (23)

where

$$P_{\left[i,j\right]}=P_{\left[i,j\right]}^{\left(1\right)}\frac{1-{\tilde{\sigma }}_{\left[i,j\right]}^{Z}}{2}+P_{\left[i,j\right]}^{\left(3\right)}\frac{1+{\tilde{\sigma }}_{\left[i,j\right]}^{Z}}{2}$$ (24)

$P_{\left[i,j\right]}^{\left(m\right)}$ is the projector to the subspace with *M*_[*i*,*j*]_ = *m*, and

$$P_{\left[i,j\right]}^{\left(m\right)}=f_{m}^{-1}\prod_{n=0}^{M_{\text{max}}}[M_{\left[i,j\right]}-(n-\delta_{m,n})1]$$ (25)

Here, *M*_max_ is the maximum number of excitations, that is

$$M_{\text{max}}=\{\begin{array}{cc} 4 & \text{if}\;i+2<j\\ 3 & \text{if}\;i+2=j \end{array}$$ (26)

and

$$f_{m}=\prod_{n=0}^{M_{\text{max}}}[m-(n-\delta_{m,n})]$$ (27)

Therefore, [*P*_0_, *H*_phys_] = 0 and *S*_[*i*,*j*]_*P*_0_ = −*P*_0_. Taking μ_*i*,*j*_ = (−1)^*i*(*j* − *i*)^, conditions (i) and (ii) are satisfied.

We take

$$U_{k}^{†}=V_{k}=\prod_{\left[i,j\right]}(\sum_{m\neq 1}P_{\left[i,j\right]}^{\left(m\right)}+P_{\left[i,j\right]}^{\left(1\right)}{\tilde{\sigma }}_{\left[i,j\right]}^{X})$$ (28)

which are unitary operators describing controlled flip operations on ancillary spins. The ancillary spin [*i*, *j*] is flipped if and only if *M*_[*i*,*j*]_ = 1. Then, we have

$$U_{k}P_{\left[i,j\right]}U_{k}^{†}=\frac{1-S_{\left[i,j\right]}}{2}\frac{1+{\tilde{\sigma }}_{\left[i,j\right]}^{Z}}{2}$$ (29)

that is, *P*_0_ is 2^*N*^-dimensional. Because

$$[\frac{1-S_{\left[i,j\right]}}{2}\frac{1+{\tilde{\sigma }}_{\left[i,j\right]}^{Z}}{2},\sigma_{k}^{X}]=0$$ (30)

we have $[P_{\left[i,j\right]},U_{k}\sigma_{k}^{X}V_{k}]=0$. Therefore, $[P_{0},U_{k}\sigma_{k}^{X}V_{k}]=0$, and condition (iii) is satisfied. When Δ is large enough, condition (iv) can be satisfied.

### Ancilla constructions for many-body stabilizers

Suppose that we wish to constrain some odd number *M* of physical spins to a given eigenvalue, + 1 or −1, of the stabilizer ${\tilde{\sigma }}_{1}^{Z}{\tilde{\sigma }}_{2}^{Z}\ldots {\tilde{\sigma }}_{M}^{Z}$. We can do so by including the following term into the constraint Hamiltonian *H*_C_

$${\left(\sum_{i=1}^{M}{\tilde{\sigma }}_{i}^{Z}\mp (I+2\sum_{j=1}^{P}{\tilde{\sigma }}_{\left[j\right]}^{Z})\right)}^{2}$$

Here, as in the main text, the square brackets [ ] in the subscript denote an ancilla, and the number of ancillas *P* is (*M* − 1)/2. The term in the inner brackets has eigenvalues *M*, *M* − 4, …, 2 − *M*. These are precisely the permitted values of the sum $\sum_{i=1}^{M}{\tilde{\sigma }}_{i}^{Z}$ if we are in a positive eigenstate of the stabilizer, so that subtracting them implies that only the acceptable states can achieve the minimum energy of this complete term (the minimum being zero since it is squared). The negative stabilizer is enforced by choosing to add rather than subtract the inner bracket for an analogous reason.

Multiplying out this term will produce ${\tilde{\sigma }}^{Z}{\tilde{\sigma }}^{Z}$ terms between the various spins involved, as well as a series of single-spin terms, which must be accounted for (in the case of the physical spins) by adjusting the on-site *J* values.

Notice that for this case of odd *M*, the same number of ancilla spins is required regardless of whether we wish to enforce a positive or a negative value for the stabilizer. If instead we wish to constrain some even number *M* of physical spins to a given eigenvalue of our stabilizer, then the number of ancillas required depends on the chosen sign. In either case, the form of the term to include in *H*_C_ is the following

$${\left(\sum_{i=1}^{M}{\tilde{\sigma }}_{i}^{Z}+2\sum_{j=1}^{P}{\tilde{\sigma }}_{\left[j\right]}^{Z}\right)}^{2}$$

However, the number of ancillas *P* is equal to *M*/2 if the stabilizer constraint is negative and *M*/2 + 1 if the constraint is positive. This is because the positive stabilizer eigenstates have *M* − 1 different possible eigenvalues of the total *z* spin, $\sum_{i=1}^{M}{\tilde{\sigma }}_{i}^{Z}$ (namely, *M*, *M* − 4, …, −*M*). Meanwhile, the negative stabilizer eigenstates have *M* − 2 possible eigenvalues of the total *z* spin (namely, *M* − 1, *M* − 5, …, 1 −*M*).

For the case of *M* = 4 spin stabilizers, following LHZ, one can use a qutrit rather than a qubit, but regardless of this choice, the ancilla structure is simpler if one opts to enforce negative stabilizers.

## References

[1] Kadowaki T., Nishimori H., Quantum annealing in the transverse Ising model. Phys. Rev. E 58, 5355 (1998).

[2] Farhi E., Goldstone J., Gutmann S., Lapan J., Lundgren A., Preda D., A quantum adiabatic evolution algorithm applied to random instances of an NP-complete problem. Science 292, 472–475 (2001). 11313487 10.1126/science.1057726

[3] Martoňák R., Santoro G., Tosatti E., Quantum annealing by the path-integral Monte Carlo method: The two-dimensional random Ising model. Phys. Rev. B 66, 094203 (2002).

[4] Kirkpatrick S. C., Gelatt C. D. Jr, Vecchi M. P., Optimization by simulated annealing. Science 220, 671–680 (1983). 17813860 10.1126/science.220.4598.671

[5] Childs A. M., Farhi E., Preskill J., Robustness of adiabatic quantum computation. Phys. Rev. A 65, 012322 (2001).

[6] Rønnow T. F., Wang Z., Job J., Boixo S., Isakov S. V., Wecker D., Martinis J. M., Lidar D. A., Troyer M., Defining and detecting quantum speedup. Science 345, 420–424 (2014). 25061205 10.1126/science.1252319

[7] Heim B., Rønnow T. F., Isakov S. V., Troyer M., Quantum versus classical annealing of Ising spin glasses. Science 348, 215–217 (2015). 25765071 10.1126/science.aaa4170

[8] Bunyk P. I., Hoskinson E. M., Johnson M. W., Tolkacheva E., Altomare F., Berkley A. J., Harris R., Hilton J. P., Lanting T., Przybysz A. J., Whittaker J., Architectural considerations in the design of a superconducting quantum annealing processor. IEEE Trans. Appl. Supercond. 24, 1–10 (2014).

[9] Jörg T., Krzakala F., Kurchan J., Maggs A. C., Pujos J., Energy gaps in quantum first-order mean-field–like transitions: The problems that quantum annealing cannot solve. Europhys. Lett. 89, 40004 (2010).

[10] Jörg T., Krzakala F., Semerjian G., Zamponi F., First-order transitions and the performance of quantum algorithms in random optimization problems. Phys. Rev. Lett. 104, 207206 (2010). 20867059 10.1103/PhysRevLett.104.207206

[11] Choi V., Minor-embedding in adiabatic quantum computation: I. The parameter setting problem. Quantum Inf. Process. 7, 193–209 (2008).

[12] Choi V., Minor-embedding in adiabatic quantum computation: II. Minor-universal graph design. Quantum Inf. Process. 10, 343–353 (2011).

[13] Boothby T., King A. D., Roy A., Fast clique minor generation in Chimera qubit connectivity graphs. Quantum Inf. Process. 15, 495–508 (2016).

[14] Cai J., Macready W. G., Roy A., A practical heuristic for finding graph minors. Quantum Phys. arXiv:1406.2741 (2014).

[15] Lechner W., Hauke P., Zoller P., A quantum annealing architecture with all-to-all connectivity from local interactions. Sci. Adv. 1, e1500838 (2015). 26601316 10.1126/sciadv.1500838 PMC4646830

[16] Albash T., Vinci W., Lidar D. A., Simulated quantum annealing with two all-to-all connectivity schemes. arXiv:1603.03755 (2016).

[17] Cheeseman P., Kanefsky B., Taylor W. M., Where the really hard problems are. IJCAI 1, 331–337 (1991).

[18] Achlioptas D., Naor A., Peres Y., Rigorous location of phase transitions in hard optimization problems. Nature 435, 759–764 (2005). 15944693 10.1038/nature03602

[19] Bravyi S. B., Kitaev A. Y., Quantum codes on a lattice with boundary. arXiv:9811052 (1998).

[20] Chancellor N., Zohren S., Warburton P. A., Circuit design for multi-body interactions in superconducting quantum annealing system with applications to a scalable architecture. arXiv:1603.09521 (2016).

[21] Leib M., Zoller P., Lechner W., A Transmon quantum annealer: Decomposing many-body Ising constraints into pair interactions. arXiv:1604.02359 (2016).

[22] Chancellor N., Zohren S., Warburton P. A., Benjamin S. C., Roberts S., A direct mapping of max k-SAT and high order parity checks to a Chimera graph. arXiv:1604.00651 (2016). 10.1038/srep37107 PMC5114552 27857179

[23] Pastawski F., Preskill J., Error correction for a proposed quantum annealing architecture. Phys. Rev. A 93, 052325 (2016).

[24] For details of the ARC service, please see http://dx.doi.org/10.5281/zenodo.22558.

